# Double-adjustment in propensity score matching analysis: choosing a threshold for considering residual imbalance

**DOI:** 10.1186/s12874-017-0338-0

**Published:** 2017-04-28

**Authors:** Tri-Long Nguyen, Gary S. Collins, Jessica Spence, Jean-Pierre Daurès, P. J. Devereaux, Paul Landais, Yannick Le Manach

**Affiliations:** 1 Laboratory of Biostatistics, Epidemiology, Clinical Research and Health Economics, UPRES EA2415, Montpellier University, Montpellier, France; 2 Departments of Anesthesia & Clinical Epidemiology and Biostatistics, Michael DeGroote School of Medicine, Faculty of Health Sciences, McMaster University and the Perioperative Research Group, Population Health Research Institute, Hamilton, Canada; 3 Centre for Statistics in Medicine, Nuffield Department of Orthopaedics, Rheumatology and Musculoskeletal Sciences, Botnar Research Centre, University of Oxford, Windmill Road, Oxford, UK; 4 Departments of Medicine, Michael DeGroote School of Medicine, Faculty of Health Sciences, McMaster University, Hamilton, Canada; 5 Department of Biostatistics, Clinical Research and Medical Informatics, Nîmes University Hospital, Nîmes, France

**Keywords:** Causal inference, Propensity score, Covariate balance, Confounding

## Abstract

**Background:**

Double-adjustment can be used to remove confounding if imbalance exists after propensity score (PS) matching. However, it is not always possible to include all covariates in adjustment. We aimed to find the optimal imbalance threshold for entering covariates into regression.

**Methods:**

We conducted a series of Monte Carlo simulations on virtual populations of 5,000 subjects. We performed PS 1:1 nearest-neighbor matching on each sample. We calculated standardized mean differences across groups to detect any remaining imbalance in the matched samples. We examined 25 thresholds (from 0.01 to 0.25, stepwise 0.01) for considering residual imbalance. The treatment effect was estimated using logistic regression that contained only those covariates considered to be unbalanced by these thresholds.

**Results:**

We showed that regression adjustment could dramatically remove residual confounding bias when it included all of the covariates with a standardized difference greater than 0.10. The additional benefit was negligible when we also adjusted for covariates with less imbalance. We found that the mean squared error of the estimates was minimized under the same conditions.

**Conclusion:**

If covariate balance is not achieved, we recommend reiterating PS modeling until standardized differences below 0.10 are achieved on most covariates. In case of remaining imbalance, a double adjustment might be worth considering.

**Electronic supplementary material:**

The online version of this article (doi:10.1186/s12874-017-0338-0) contains supplementary material, which is available to authorized users.

## Background

Propensity score (PS) matching analysis is a popular method for estimating the treatment effect in observational studies [1–3]. Defined as the conditional probability of receiving the treatment of interest given a set of confounders, the PS aims to balance confounding covariates across treatment groups [4]. Under the assumption of no unmeasured confounders, treated and control units with the same PS are matched, removing confounding and allowing an unbiased estimation of the treatment effect [4].

Approximating completely randomized experiment, a fundamental step in PS matching analysis is to ensure that the covariate balance across the treatment groups is achieved, by using diagnostics that have been described in the literature [5, 6]. However, King and Nielsen showed that PS matching was likely to be concerned by covariates imbalance [7]. If balance is achieved across all of the confounders, the treatment effect can be estimated without bias. If balance is not possible, PS models can be re-specified until a correct balance is achieved. In a next step, any unbalanced covariates can be adjusted within the PS-matched sample [8].

Although arbitrary thresholds for standardized differences have been proposed for detecting residual imbalance across groups in matched samples [9], there is no consensus on which threshold value should be used to choose the covariates for regression adjustment. If a sample is large enough to contain sufficient outcomes [10–12], all of the covariates can be adjusted. However, small samples, which are more likely to suffer imbalance, limit the number of covariates that can be included, and specifying criterion strict enough to remove sufficient residual confounding is problematic. We hypothesize that a threshold would have to be respected to ensure unbiased estimate of treated effect.

As not all covariates can be adjusted, we aimed to determine the optimal imbalance threshold for choosing the covariates for regression adjustment to remove residual confounding. The threshold should be the highest tolerable standardized difference that does not compromise treatment effect estimation.

## Methods

### Data generation

We conducted a series of Monte Carlo experiments based on simulated data sets that mimicked real clinical settings in the perioperative field [13, 14], by using an approach similar to Setoguchi’s method [15]. We designed 15 explanatory variables (*W*_*1*_ to *W*_*15*_) by generating a set of 14 normal random variables correlated by different degrees (Additional file 1: Figure S1) and adjusting and dichotomizing them to obtain distributions similar to real perioperative variables, (Additional file 1: Table S1). Of these, some were defined as continuous variables with distributions approximating biological markers, while others were binary variables, the prevalence of which approximated comorbidities reported in perioperative studies (Additional file 1: Table S1). One of the covariates (*W*_*14*_) did not follow this generation process, but was defined as a combination of the others, mimicking the revised cardiac risk index of Lee et al. [16]. We generated a binary treatment variable *Z* (*Z* = 1 denotes treated units, *Z* = 0 denotes control units) and a binary outcome variable *Y* (*Y* = 1 denotes the occurrence, *Y* = 0 denotes the non-occurrence). Logistic models were used for treatment assignment (*i.e.* the true PS models) and generating the outcome. Two scenarios were designed:

Scenario A – linearity and additivity:

$$ logit\left[ p(Z)\right] = {\beta}_0+{\beta}_1{W}_1+{\beta}_2{W}_2+{\beta}_6{W}_6+{\beta}_7{W}_7+{\beta}_{11}{W}_{11}+{\beta}_{13}{W}_{13} $$

$$ l o g i t\left[ p(Y)\right]={\alpha}_0+{\alpha}_1{W}_1+{\alpha}_2{W}_2+{\alpha}_3{W}_3+{\alpha}_4{W}_4+{\alpha}_5{W}_5+{\alpha}_7{W}_7+{\alpha}_9{W}_9+{\alpha}_{10}{W}_{10}+{\alpha}_{11}{W}_{11}+{\alpha}_{12}{W}_{12}+{\alpha}_{13}{W}_{13}+{\alpha}_{15}{W}_{15}+{\gamma}_Z Z $$

Scenario B – non-linearity and non-additivity:

$$ logit\left[ p(Z)\right] = {\beta}_0+{\beta}_1{W}_1+{\beta}_2{W}_2+{\beta}_6{W}_6+{\beta}_7{W}_7+{\beta}_{11}\left({W_{11}}^{1/2} + 0.01\ {W_{11}}^2\right) + {\beta}_{13}{\left({W}_{13}\right)}^{1/2}+{\beta}_1(0.4){W}_1{W}_2+{\beta}_7(0.5){W}_7{W}_1+{\beta}_2(0.7){W}_7{W}_2+{\beta}_{11}(0.7){\left({W}_{11}\right)}^{1/2}\left({W}_{13}/10\right) $$

$$ logit\left[ p(Y)\right] = {\alpha}_0+{\alpha}_1{W}_1+{\alpha}_2{W}_2+{\alpha}_3{W}_3+{\alpha}_4{W}_4+{\alpha}_5{W}_5+{\alpha}_7{W}_7+{\alpha}_9{W}_9+{\alpha}_{10}{W}_{10}+{\alpha}_{11} Log\left({W}_{11}\right) + {\alpha}_{12}{\left({W}_{12}\right)}^{1/2}+{\alpha}_{13}{\left({W}_{13}\right)}^{1/3}+{\alpha}_{15}{W}_{15}+{\gamma}_Z Z + {\alpha}_{10}(0.2){W}_{10}{W}_7+{\alpha}_4(0.7){W}_4{W}_2+{\alpha}_1(0.6){W}_1{W}_3{W}_7 $$

We set the treatment exposure at 40% of the population and the outcome prevalence at 8%. Treatment assignment (outcome occurrence) was assigned if p(Z) (p(Y)) was greater than a randomly U(0,1) generated number. We report the *βi* and *αi* coefficients used for this simulation in Additional file 1: Table S1. According to the equations, we considered two noise-variables (*W*_*8*_ and *W*_*14*_) which were nonetheless correlated to the others (Additional file 1: Figure S1) and as such, at risk of imbalance.

The treatment effect was defined to be protective (coefficient *γ*_*T*_ = −0.51, conditional odds ratio = 0.60), in alignment with published perioperative studies [17–19]. As PS analysis allows a marginal effect to be calculated, conditional and marginal effects were to differentiate. Conditional effect refers to the mean of every subject-specific effects, while marginal effect refers to the average effect that would be observed if the overall population were to be treated (*versus* if it were to be untreated). This marginal effect can be measured, as said in the overall population (*i.e*. the average treatment effect, ATE), or in the subpopulation in which the treatment was intended (*i.e.* the average treatment effect in the treated, ATT). This ATT is the estimand of PS matching analysis. In our simulations, the true ATT was −0.04 on the absolute risk difference scale. We simulated *N* = 1,000 samples, comprising 5,000 units each. This sample size provided sufficient outcomes for performing reliable regression adjustments after matching [10, 11], as described in the following subsection.

### Propensity score matching analysis

We derived a PS model for each sample from a logistic regression that included all of the *W*_*i*_ covariates. No interaction terms were used. It has been recognized that including instrumental variables inflates the bias [20–22], so this model is believed to be realistic [1, 3] rather than optimal. Treated and control units were then matched according to their estimated PS using a 1:1 ratio without replacement [23, 24]. Though using a caliper in PS matching is common, this practice might lead to incomplete matching by discarding subjects for whom no matches are found within the distance imposed by the caliper. Doing so removes confounding, at the expense of reducing the matched sample. For this reason, we used a nearest-neighbor matching algorithm without a caliper. We evaluated the balance of each covariate *W*_*i*_ across the treatment groups in each matched sample by calculating the standardized absolute mean difference [5, 6]:

$$ S M D=\frac{\left|\overline{W_{\iota 1}}-\overline{W_{\iota 0}}\right|}{\sqrt{\frac{S_{i1}^2+{S}_{i0}^2}{2}}} $$

$\overline{W_{\iota 1}}$ and $\overline{W_{\iota 0}}$ denote the means (proportions for discrete variables), and *s*_*i*1_^2^ and *s*_*i*0_^2^ denote the variances in the treated and control groups, respectively. We entered the unbalanced covariates into second logistic models to remove residual confounding before estimating the treatment effect, a process called the double-robust estimator. As there is as yet no consensus on the SMD threshold, we tested a set of SMD values for choosing the covariates for use in logistic regression. We defined 25 SMD thresholds from 0.01 to 0.25, increasing stepwise by 0.01. We computed 25 double-robust estimates of the ATT per matched sample, one with each threshold. To this end, we performed regressions in a way akin to Abadie and Imbens [8]. As noted by Austin P.C. [25], it is important to distinguish between traditional adjustment that estimates conditional effect and this method of double-adjustment that estimates marginal effect. To perform double-adjustment, we fitted two logistic models within each arm of each matched sample. The models included the outcome *Y* as the dependent variable and all of the unbalanced covariates as explanatory variables, chosen using the SMD threshold value.

From those to regressions, let $\widehat{p}\left({Y}_1=1\Big| Z=1\right)$ denote the predicted outcome probability in the treated group according to the model derived on the treated arm (*i.e.* the potential outcome with treatment). Let $\widehat{p}\left({Y}_0=1\Big| Z=1\right)$ denote the predicted outcome probability in the treated group according to the model derived on the control arm (*i.e.* the potential outcome without treatment). Let *j* index the treated matched unit (the pair, in case of 1:1 matching). The double-robust ATT estimator can be calculated as:

$$ {\widehat{ATT}}_{DR}=\frac{1}{J}{\displaystyle \underset{j=1}{\overset{J}{\Sigma}}}\widehat{p_J}\left({Y}_1=1\Big| Z=1\right)\mathit{\hbox{-}}\frac{1}{J}{\displaystyle \underset{j=1}{\overset{J}{\Sigma}}}\widehat{p_J}\left({Y}_0=1\Big| Z=1\right)=\frac{1}{J}{\displaystyle \underset{j=1}{\overset{J}{\Sigma}}}\left[\widehat{p_J}\left({Y}_1=1\Big| Z=1\right) - \widehat{p_J}\left({Y}_0=1\Big| Z=1\right)\right] $$

We note that, in case of non-collapsible effect (*e.g*. odds ratio), one can still estimate a marginal effect (*e.g*. $\frac{\left[\frac{1}{J}{\displaystyle {\varSigma}_{j=1}^J}\widehat{p_j}\left({Y}_1=1\Big| Z=1\right)\right]/\left[1-\frac{1}{J}{\displaystyle {\varSigma}_{j=1}^J}\widehat{p_j}\left({Y}_1=1\Big| Z=1\right)\right]}{\left[\frac{1}{J}{\displaystyle {\varSigma}_{j=1}^J}\widehat{p_j}\left({Y}_0=1\Big| Z=1\right)\right]/\left[1-\frac{1}{J}{\displaystyle {\varSigma}_{j=1}^J}\widehat{p_j}\left({Y}_0=1\Big| Z=1\right)\right]}$, for estimating an odds ratio).

We also estimated the ATT on the matched samples without the double-robust approach, using a crude estimator:

$$ {\widehat{ATT}}_{crude}=\frac{1}{J}{\displaystyle \sum_{j=1}^J}\left({Y}_{j1}-{Y}_{j0}\right) $$

We report the relative bias and mean squared error for each estimator:

$$ Relative\kern0.5em  bias\kern0.5em \left(\%\right)=100\times \frac{\left|\widehat{ AT T}- AT{T}_{true}\right|}{AT{T}_{true}} $$

$$ M S E=\frac{1}{N}{\displaystyle \underset{n=1}{\overset{N}{\varSigma}}}{\left(\widehat{ATT}- AT{T}_{true}\right)}^2 $$

## Results

**Fig. 1:**
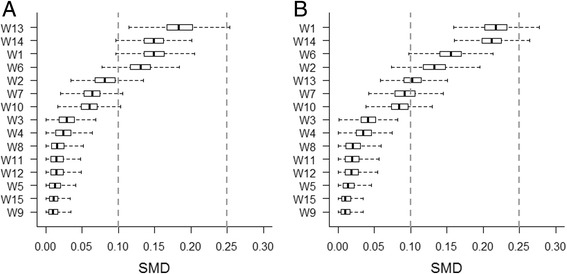
Covariate balance diagnostics after nearest-neighbor matching in (**a**) a linear, additive scenario and (**b**) a non-linear, non-additive scenario. SMD, standardized absolute mean difference

Performing 1:1 nearest-neighbor matching resulted in keeping, on average, 80.0% and 80.2% of the subjects from the initial samples in scenarios A and B, respectively. The majority of the true confounders in both scenarios were not consistently well-balanced (Fig. 1). This situation was expected, as it was required to evaluate the performance of the double-robust approach for removing residual confounding bias.

**Fig. 2:**
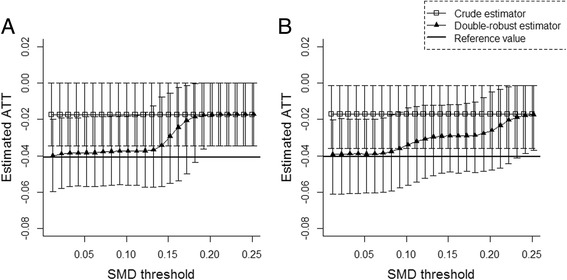
Estimated average treatment effect in the treated (ATT) on the absolute risk difference scale (median with 2.5^th^ and 97.5^th^ percentiles), in (**a**) the linear, additive scenario and (**b**) the non-linear, non-additive scenario. The double-robust estimator was adjusted for unbalanced covariates using standardized absolute mean difference (SMD) thresholds

Figure 2 shows that the crude estimator was systematically biased. The correction provided by the double-robust approach varied with the SMD threshold value used to choose the unbalanced covariates for regression adjustment. As expected, bias correction was more successful with stricter thresholds. The relationship between the bias reduction and the SMD threshold was sigmoidal. In both scenarios, very little confounding bias remained after adjusting for all covariates with approximately SMD > 0.10. Adjusting for covariates with SMD > 0.10 resulted in a substantial bias reduction, while adjusting for covariates with less imbalance did not lead to a major improvement in the estimation.

**Fig. 3:**
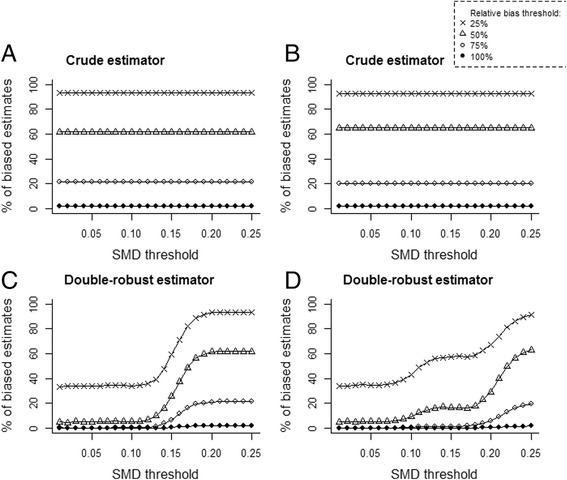
Percentage of biased estimates according to the estimator in (**a** and **c**) the linear, additive scenario and (**b** and **d**) the non-linear, non-additive scenario. The double-robust estimator was adjusted for unbalanced covariates using standardized absolute mean difference (SMD) thresholds

We calculated the relative bias and the percentage of estimates that differed from the true ATT by 25%, 50%, 75% and 100% (Fig. 3). The double-robust approach dramatically reduced the percentage of biased estimates. This percentage was minimized when covariates with an SMD equal to or greater than 0.10 were adjusted.

**Fig. 4:**
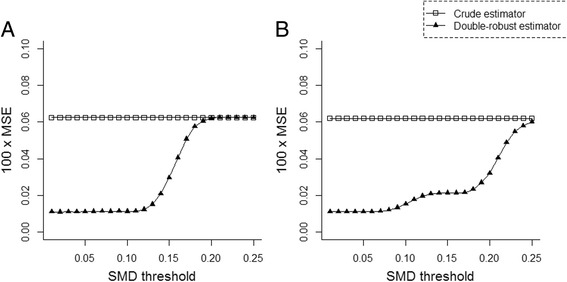
Mean squared error according to the estimator in (**a**) the linear, additive scenario, and (**b**) the non-linear and non-additive scenario. The double-robust estimator was adjusted for unbalanced covariates using standardized absolute mean difference (SMD) thresholds

Adjusting for unbalanced covariates also improved the estimator’s performance, as measured by the mean squared error (Fig. 4). Again, a threshold of SMD approximately equal to 0.10 minimized the mean squared error.

## Discussion

We showed that treatment effect estimation was compromised when an SMD greater than 0.10 remained in a data set and that performing double-adjustment on PS-matched samples addressed the issue of residual confounding bias. We suggest that at least all covariates with SMD > 0.10 should be adjusted for to ensure an unbiased estimate.

We have shown the importance of balance diagnostics after matching. PS matching methods aim to remove confounding bias by balancing covariates across treatment groups using a balancing score. Although arbitrary thresholds have been proposed for detecting residual imbalance, some authors have recommended removing all imbalance [26, 27], which is of greatest priority in PS analysis. In case of imbalance, several strategies are to consider before performing an adjustment, which include re-specifying the PS model until a correct balance is achieved or using other designs, such as stratification, weighting or full matching [28–31]. When the covariate balance cannot be achieved in spite of these considerations, adjusting for unbalanced covariates within the matched sample is an attractive approach. As we showed, the matched sample’s balance diagnostics can be used as a threshold for performing double-adjustment on it. The benefit provided by the correction proposed by Abadie and Imbens [8] depended on the magnitude of the imbalance in the covariates we adjusted for. We showed that adjusting for highly unbalanced covariates substantially reduced residual confounding, whereas adjusting for weakly unbalanced covariates did not result in a major correction. We minimized the residual confounding bias in both studied scenarios when we adjusted for all covariates with SMD > 0.10.

We support previous statements that recommend removing all imbalance [26, 27]. In contrast with coarsened exact matching, PS matching is likely to be concerned by imbalance [7]. However, adjusting for all covariates may not be possible in small matched samples, as their few endpoints may limit regression [10–12], yet small matched-samples are particularly affected by residual imbalance. We suggest that, at minimum, all covariates with SMD > 0.10 should be adjusted in a small sample. This threshold corresponds to the limit of tolerable imbalance that does not compromise treatment effect estimation. If the sample contains sufficient outcomes, additional covariates can also be adjusted for to remove the remaining residual confounding.

We assessed imbalance using the SMD. This measure has previously been proposed for assessing covariate balance across groups [6]. It depicts the balance property of the sample and does not depend on its size, as suggested by Imai, King and Stuart [27]. According to recent systematic reviews, this metric is still poorly implemented in practice, as inferential tests are wrongly used instead [1, 3]. We support the evaluation of covariate balance using appropriate metrics such as the SMD. Recent weighted balance metrics have been described in the literature [32, 33], the advantage of which is to weight the SMD by the strength of the association between each covariate and the outcome. They hypothesized that residual confounding bias is more important when strong confounders are unbalanced than when other covariates are unbalanced. In a future study, we will assess whether these metrics improve the bias-correction provided by our double-robust approach.

Although it is challenging, a PS model’s specifications should be improved before a regression adjustment is systematically conducted on the PS-matched samples. This step reduces imbalance across groups and thus the number of covariates that need to be adjusted. It also improves the overlapping value of the estimated PS between the treated and control units, which can increase the number of pairs and the matched sample size if a caliper is used. In this simulation study, we did not re-specify PS models, since we sought to explore at which threshold of imbalance double-adjustment might be worth considering. We emphasize that achieving covariate balance avoids the need for double-adjustment, which is a model-dependent approach. As the estimated effect varies with the model’s specification, the analyst is likely to be tempted to report the result, which fit its “favorite hypothesis” [7, 34]. For that reason, King and Nielsen advocate the use of other matching methods [7].

Our study should be interpreted in light of some limitations. We only used a PS matching algorithm without caliper, the use of which has been recommended [24]. Though caliper matching reduces imbalance, it can result in incomplete matching [35]. It only keeps treated subjects with a PS close enough to a control unit to be matched, resulting in the exclusion of units. As pointed out by Austin, double-adjustment should be used to handle residual bias only after complete matching [25]. Because using caliper reduces imbalance, we hypothesize that it will diminish the benefit of double-adjustment, which should be explored in a further study. Although matching on the PS has been criticized recently [7], we performed a 1:1 nearest-neighbor algorithm because we believe that this is reflective of current practices [1, 3]. A recent study comparing 12 matching algorithms also did not find substantial differences in balancing by nearest-neighbor matching and other algorithms such as optimal matching [24]. Additionally, double-adjustment involves modeling steps and is thus exposed to the risk of model dependence. We did not used the double-robust estimator proposed by Austin [25], which adjusts for the estimated PS rather than for the baseline covariates. We hypothesize that any misspecification in the PS would negatively affect this estimator. However, this interesting method may be useful if the sample size limits the inclusion of covariates into regression adjustment. In a future study, we will compare the performance of these two bias-corrected estimators within additional scenarios of misspecification and apply such approaches to real clinical data.

## Conclusions

We support the reporting of balance diagnostics for PS-matched samples. Measures like the SMD can be used as a criterion for choosing the covariates for double-adjustment. This approach addresses the issue of residual confounding in treatment effect estimation. If the sample is large enough, all of the covariates can be added into a regression and adjusted. In small samples, we suggest at least adjusting for those covariates with an SMD equal to or greater than 0.10 to remove imbalance that can comprise the reliability of the treatment effect estimation.

## Additional file

Additional file 1: ‘Supplementary material: simulation design’. Variable definitions and coefficients for data generation (**Table S1** and **Table S2**), and variable relationships and correlations (**Figure S1**). (DOCX 143 kb)

## Abbreviations

ATT: Average treatment effect in the treated
MSE: Mean squared error
PS: Propensity score
SMD: Standardized mean difference
