# Supplementary material for: Double-adjustment in propensity score matching analysis: choosing a threshold for considering residual imbalance
Source: BMC Med Res Methodol. 2017 Apr 28;17:78. doi: 10.1186/s12874-017-0338-0 (PMC5408373; doi:10.1186/s12874-017-0338-0)

**Double-adjustment in propensity score matching analysis: choosing a threshold for considering residual imbalance.**

***Supplementary material: simulation design.***

**Table S1.** Variable definitions and coefficients for data generation. The *α* and *β* coefficients define the true outcome model and true propensity score model, respectively.

**Scenario A: linearity and additivity**

| **Variable name** | **Variable type** | **Related perioperative variable** | *α coefficients* | *β coefficients* |
| --- | --- | --- | --- | --- |
| *Intercept* | | | -3.3 | 0.16 |
| *W_1_* | Binary | *Coronary artery disease* | 0.70 | 0.70 |
| *W_2_* | Binary | *Chronic renal failure* | 0.64 | 0.63 |
| *W_3_* | Binary | *Diabetes* | 0.31 | 0 |
| *W_4_* | Binary | *Chronic heart failure* | 0.80 | 0 |
| *W_5_* | Binary | *Chronic obstructive pulmonary disease* | 0.54 | 0 |
| *W_6_* | Binary | *History of stroke* | 0 | 0.65 |
| *W_7_* | Binary | *Hypertension* | 0.28 | 0.27 |
| *W_8_* | Binary | *Obesity* | 0 | 0 |
| *W_9_* | Binary | *History of cancer* | 0.64 | 0 |
| *W_10_* | Binary | *Peripheral vascular disease* | 0.75 | 0 |
| *W_11_* | Continuous | *Age (per year)* | 0.02 | 0.02 |
| *W_12_* | Continuous | *Preoperative hemoglobin (per g/dL)* | 0.05 | 0 |
| *W_13_* | Continuous | *Preoperative eGFR (per mL/min)* | -0.03 | -0.03 |
| *W_14_* | Ordinal  (5 levels) | *Revised cardiac risk index (per level)* | 0 | 0 |
| *W_15_* | Ordinal  (3 levels) | *Type of surgical procedure (per level)* | 0.59 | 0 |

**Scenario B: non-linearity and non-additivity**

| **Variable name** | **Variable type** | **Related perioperative variable** | *α coefficients* | *β coefficients* |
| --- | --- | --- | --- | --- |
| *Intercept* | | | -1.48 | 1.48 |
| *W_1_* | Binary | *Coronary artery disease* | 0.77 | 0.75 |
| *W_2_* | Binary | *Chronic renal failure* | 0.65 | 0.70 |
| *W_3_* | Binary | *Diabetes* | 0.21 | 0 |
| *W_4_* | Binary | *Chronic heart failure* | 0.34 | 0 |
| *W_5_* | Binary | *Chronic obstructive pulmonary disease* | 0.24 | 0 |
| *W_6_* | Binary | *History of stroke* | 0 | 0.65 |
| *W_7_* | Binary | *Hypertension* | 0.54 | 0.60 |
| *W_8_* | Binary | *Obesity* | 0 | 0 |
| *W_9_* | Binary | *History of cancer* | 0.64 | 0 |
| *W_10_* | Binary | *Peripheral vascular disease* | 0.55 | 0 |
| *W_11_* | Continuous | *Age (per year)* | 0.18 | 0.01 |
| *W_12_* | Continuous | *Preoperative hemoglobin (per g/dL)* | 0.10 | 0 |
| *W_13_* | Continuous | *Preoperative eGFR (per mL/min)* | -0.60 | -0.41 |
| *W_14_* | Ordinal  (5 levels) | *Revised cardiac risk index (per level)* | 0 | 0 |
| *W_15_* | Ordinal  (3 levels) | *Type of surgical procedure (per level)* | 0.19 | 0 |

**Figure S1.** Variable definitions and data generation of virtual populations. We used five true confounders (*W_1_*, *W_2_*, *W_7_*, *W_11_* and *W_13_*), seven outcome predictors (*W_3_*, *W_4_*, *W_5_*, *W_9_*, *W_10_*, *W_12_* and *W_15_*), one treatment predictor (*W_6_*) and two unrelated variables (*W_8_*, *W_14_*). Arrows represent causal effects. Arcs represent correlations and are accompanied by the correlation coefficients.


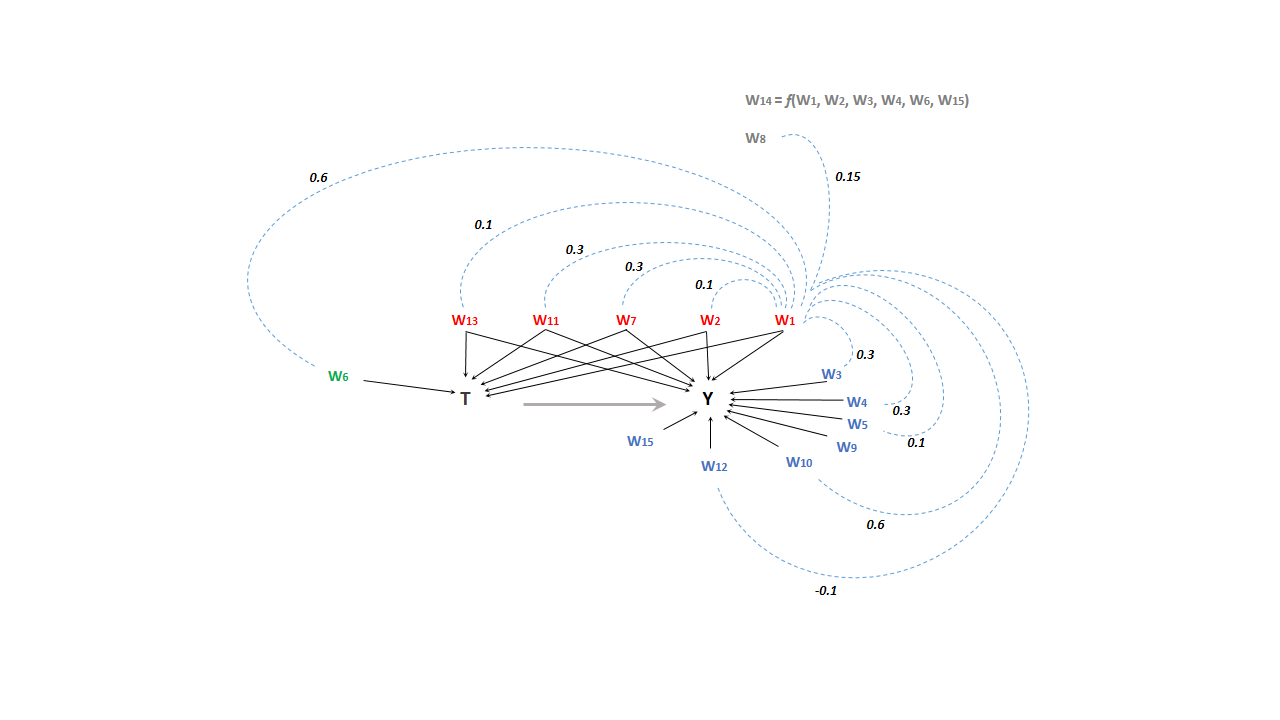

Supplement: Supplementary file 1 — ‘Supplementary material: simulation design’. Variable definitions and coefficients for data generation (Table S1 and Table S2), and variable relationships and correlations (Figure S1). (DOCX 143 kb) [file 12874_2017_338_MOESM1_ESM.docx]
